# Multi-criteria assessment to screen climate smart rice establishment techniques in coastal rice production system of India

**DOI:** 10.3389/fpls.2023.1130545

**Published:** 2023-04-18

**Authors:** Kiran Kumar Mohapatra, A. K. Nayak, R. K. Patra, Rahul Tripathi, Chinmaya Kumar Swain, K. C. Moharana, Anjani Kumar, Mohammad Shahid, Sangita Mohanty, Saheed Garnaik, Hari Sankar Nayak, Simran Mohapatra, Udaya Sekhar Nagothu, M. Tesfai

**Affiliations:** ^1^ Indian Council of Agricultural Research (ICAR) -National Rice Research Institute, Odisha, India; ^2^ Odisha University of Agriculture and Technology, Odisha, India; ^3^ Cornell University, Ithaca, NY, United States; ^4^ Norwegian Institute of Bioeconomy Research, Oslo, Norway

**Keywords:** rice production techniques, energy budget, global warming potential, climate smart index, nutrient use efficiency

## Abstract

**Introduction:**

Conventional rice production techniques are less economical and more vulnerable to sustainable utilization of farm resources as well as significantly contributed GHGs to atmosphere.

**Methods:**

In order to assess the best rice production system for coastal areas, six rice production techniques were evaluated, including SRI-AWD (system of rice intensification with alternate wetting and drying (AWD)), DSR-CF (direct seeded rice with continuous flooding (CF)), DSR-AWD (direct seeded rice with AWD), TPR-CF (transplanted rice with CF), TPR-AWD (transplanted rice with AWD), and FPR-CF (farmer practice with CF). The performance of these technologies was assessed using indicators such as rice productivity, energy balance, GWP (global warming potential), soil health indicators, and profitability. Finally, using these indicators, a climate smartness index (CSI) was calculated.

**Results and discussion:**

Rice grown with SRI-AWD method had 54.8 % higher CSI over FPR-CF, and also give 24.5 to 28.3% higher CSI for DSR and TPR as well. There evaluations based on the climate smartness index can provide cleaner and more sustainable rice production and can be used as guiding principle for policy makers.

## 1 Introduction

Sustainable rice cultivation in Asia is important for global food security, as rice fulfills the calorie requirement of more than 50% of the world population. As per the future projection, the rice production should be increased by 30% to meet out the future food demand (Pathak et al., 2020). Currently, rice is grown on 43.79 million hectares (Mha) of cultivated land in India, with a production of 116.4 million tons (Mt) (GOI, 2020). Farmers often wet till their paddies to transplant young seedlings of rice. Tillage at inappropriate moisture destructs the soil structure and declines the soil health over the long run to support a healthy crop (Altieri, 2018). In addition, rice is blamed for its high-water requirement and greenhouse gas emission due to its cultivation in completely saturated and flooded condition. The water footprint of rice production in India is 2,020 M^3^ a year compared with 970 M^3^ a year in China and a global average of 1,325 M^3^ a year (Bouraima et al., 2015). Rice requires 800–5,000 L of water with an average of 2,500 L/kg of grain yield (Bouman and Tuong, 2000; Najmuddin et al., 2018), and the water productivity of rice ranges between 0.20 and 1.20 kg m^−3^, which is one of the least among the cereal crops with equal caloric requirements (Najmuddin et al., 2018; Kumar et al., 2019). Globally, rice fields account approximately 30% and 11% of agricultural emissions of CH_4_ and N_2_O, respectively (Hussain et al., 2015). In India, rice fields emit 4.09 ± 1.19 Tg CH_4_ year^−1^ of methane, which is approximately 5% of the total methane emission from agriculture (Gupta et al., 2021). Thus, in the era of climate change and energy crisis, focusing and popularizing the sustainable crop production practices are the needs of the hour. The crop production should be associated with minimum use of water, energy, fertilizer, and other farm inputs to reduce the environmental foot print associated with rice production

In past decades, alternative rice establishment techniques have been advocated for sustainable intensification of rice production systems (Jat et al., 2019), namely, system of rice intensification (SRI), direct seeded rice (DSR), line transplanting, alternate wetting and drying (AWD), etc. These techniques are popularly called as resource conservation techniques (RCTs), as they improve the use efficiency of applied resources. SRI has been adopted by more than 10 million farmers in 70 or more countries (Thakur et al., 2016). Rice grown under SRI methods has merit of improving resource use efficiency and crop productivity while mitigating greenhouse gas (GHG) emissions (Gathorne-Hardy, 2013). Adaptation of SRI methods reduced 28%–30% global warming potential (GWP) and 36% water use over the farmer practice (Jain et al., 2014). DSR rice production is another popular crop establishment technique with coverage of 29 Mha in Asia (Pathak et al., 2011). The main advantage of DSR rice production techniques is 10%–30% saving of irrigation water (Kumar et al., 2022; Rana et al., 2022), reduction in GWP by 16%–33% (Pathak and Aggarwal, 2012; Chakraborty et al., 2017; Susilawati et al., 2019), and reduction in the production cost by 6%–32% (Kumar and Ladha, 2011). The alternate wet and dry method of irrigation is usually followed along with SRI and DSR establishment techniques (Rao et al., 2017). The mechanized line transplanting of the seedlings is often advised for better stand establishment as compared to random transplanting in the farmer’s field. The benefits of mechanized transplanting are higher yield and nutrient and water use efficiency.

Although there are many studies evaluating the benefits of RCTs for crop productivity and profitability, studies evaluating multi-criteria assessment of different RCTs for sustainability indicators are very few (Kakraliya et al., 2018). Again, most of the studies have been conducted on the research stations, which does not account for the socioeconomic conditions of farmers. The on-farm assessment of crop production techniques for multiple indicators of rice production is more important (Nayak et al., 2022). Nowadays, along with productivity and profitability, the environmental footprints are more focused due to the environmental pollution/footprints associated with crop production (Sapkota et al., 2019). Common rice production/crop establishment techniques are evaluated for GHG emission, energy, fertilizer, and water use, and soil health (Parihar et al., 2019). Thus, in order to select a cleaner, efficient, and profitable rice production practices, which are climate resilient, a comprehensive approach is required. For this, the development of a common index combining the effect of multiple indicators can be more appropriate. Although many researchers have proposed the use of numerical index for evaluating climate smart agriculture practices (Arenas-Calle et al. (2019); Arenas-Calle et al. (2021)); Challinor et al., 2022), those were less comprehensive (not based on three principles of climate smart agriculture). There is no single available study assessing the performance of rice production techniques against crop and water productivity, soil health, nutrient use efficiency, energy productivity, GHG emission, and profitability, in totality based on three principles of climate smart agriculture (production, adaptation, and mitigation). In this connection, there lies an excellent opportunity to develop a unique index to compare multiple criteria at one go while screening the most efficient rice production techniques. Therefore, a 2-year experiment was conducted with the following objectives: i) to measure or compute multiple indicators of sustainability for diversified rice establishment techniques in the eastern coastal rice production system of India, ii) to identify the important soil properties affecting productivity and greenhouse gas emission from the soil, and iii) to develop an index to represent multiple indicators of sustainability (climate smartness index) to screen for a cleaner, profitable, and resource-efficient rice establishment technique. We hypothesized that the SRI techniques of rice production are more sustainable, with minimum resource use and ecological impact.

## 2 Materials and methods

### 2.1 Experimental site characteristics

A field experiment was conducted in a farmer’s field in Tangi-Choudwar block of Cuttack districts of Odisha state, India during the winter season of 2020 and 2021. This area is located at mid-central table land zone (20°66′ N latitudes and 85° 95 E′ longitudes) of Odisha and receives a mean annual rainfall of 1,400 mm. Soil type was sandy clay loam (Bouyoucos, 1962) with 1.44 Mg m^−3^ bulk density, 0.29 dS m^−1^ electrical conductivity (EC), 5.7–5.9 pH (using 1:2.5, soil:water suspension) (Jackson, 1973), 5.8 g kg^−1^ organic C (Walkley and Black, 1934), 176.5 kg ha^−1^ available N (Subbiah and Asija, 1956), 15.8 kg ha^−1^ available P (Olsen, 1954), and 87.8 kg ha^−1^ NH_4_OAc-K (Jackson, 1973) content, respectively.

### 2.2 Details of the treatment and crop management

The experiment was carried out in a randomized completely block design (RCBD) replicated four times. The rice cultivar Pooja of 130–135days duration with 5.5–6 t ha^−1^ of yield potential was cultivated during both years. Five climate-resilient rice production techniques along with farmer regular practice as control were evaluated. The treatment detail of each rice production technique has been described in **Table 1**. The treatments included I) system of rice intensification with alternate wetting and drying (SRI-AWD), II) direct seeded rice with continuous flooding (DSR-CF), III) direct seeded rice with alternate wetting and drying (DSR-AWD), IV) transplanted rice with continuous flooding (TPR-CF), V) transplanted rice with alternate wetting and drying (TPR-AWD), and VI) farmer’s practice (FPR-CF). The land was prepared once with mold board plow up to 20 cm, followed by harrowing and leveling. Each plots (100 m^2^) were isolated by bunds (30×50 cm^2^ with polythene sheets up to a depth of 45 cm to prevent interplot seepage. The treatments details related to seed rate, method of transplanting, seedling age, irrigation water management, and amount of fertilizer applied were presented in **Table 1**. Urea, di-ammonium phosphate (DAP) and muriate of potash (MOP) was applied to supply the NPK to the rice. Half dose of N and full dose of P and K were applied as basal, and the remaining half-dose of N was applied at maximum tillering stage and panicle initiation stage in two equal splits. In both DSR and transplanted condition after 1 week of showing/transplanting, 5 cm irrigation was applied when soil moisture tension dropped to −20 kPa in alternate wetting and drying (AWD) treatments throughout the rice growing season, excluding the flowering period. In conventional flooding (CF) conditions, irrigation was regularly applied to maintain a flooding condition from the day of transplanting to physiological maturity. The grain and biological yield were recorded after harvesting at harvest maturity of the crops. The grain yield was recorded at 14% moisture content. The harvest index (HI) was calculated as:

**Table 1:** Details description of different rice production techniques.

Inputs details	SRI-AWD	DSR-CF	DSR-AWD	TPR-CF	TPR-AWD	FPR-CF
Seed rate (kg ha^−1^)	5 kg	30 kg	30 kg	25 kg	30 kg	50 kg
Method of trans planting/showing	Manual transplanting	Drum seeder	Seed drill	Mechanical transplanter	Manual transplanting	Manual transplanting
Spacing (cm)	25 x 25	20 x 20	20 ×20	25x20	20 x 20	Random
Seedling age (days)	12 days	Direct sprouted seed sowing	Direct seed sowing	18 days	21 days	28 days
Seedling hill^−1^	1	–	–	1–2	2	2–3
Water management	AWD	CF	AWD	CF	AWD	CF
Rate of manure	10 Mg ha^−1^					
Fertilizer rate						
N (kg ha^−1^)	50	100	100	100	100	100
P_2_O_5_ (kg ha^−1^)	25	50	50	50	50	50
K_2_O (kg ha^−1^)	25	50	50	50	50	50
Method of harvesting	Manual harvesting	Manual harvesting	Manual harvesting	Manual harvesting	Manual harvesting	Manual harvesting

SRI, system of rice intensification; DSR, direct seeded rice; TPR, transplanted rice FPR, farmers’ practice rice; AWD, alternate wetting and drying; CF, continuous flooding.


(1) $$Harvest\;index\;\left(\%\right)=\;\frac{\text{Economic yield}}{\text{total biological yield}}\times 100$$

### 2.3 Irrigation water productivity and nitrogen partial factor productivity

The amount of applied irrigation water was measured by measuring the discharge of Parshall flume installed at the field multiplied by the water application duration (Parshall, 1928). Furthermore, the irrigation water productivity was measured across the treatments using **Equation 2**.


(2) $$\begin{array}{c} Irrigation\;water\;productivity\;\left({\text{kg m}}^{-\;3}\right)\\ =\;\frac{\text{Grain yield }\left(\text{kg}\right)}{\text{Quantity of water supply }\left(m^{3}\right)}\times 100 \end{array}$$

Nitrogen partial factor productivity (NPFP) was calculated by using **Equation 3** as described in Dobermann (2007).


(3) $$PFPN=\;\frac{\text{Grain yield }\left(\text{kg}\right)}{\text{Quantity of nitrogenous fertilizer supply }\left(\text{kg}\right)}$$

### 2.4 Energy budgeting

The consumption of input energy from the use of all the farm input and agricultural activities and energy output from rice grain, and straw yield were included for energy budgeting (Scholz, 1997; Sørensen et al., 2014). At first, the inventory of input use in all the agricultural operations, such as tillage, sowing, weeding, fertilizer, and agrochemical applications, harvesting, and threshing, was built. Furthermore, the amount of fertilizers, seeds, agrochemicals, human labor, diesel, and irrigation water were multiplied with their respective energy conversion coefficients (**Table 2**) for the calculation of input energy for different treatments. To determine the system’s energy efficiency, the following parameters were taken and calculated (Chaudhary et al., 2017) using the following equation:

**Table 2:** Energy equivalents and price of inputs and outputs in agricultural production.

Particulars	Unit	Energy equivalent (MJ unit^−1^)	Reference	Unit	Price (Rupees)/unit
Inputs					
Human labour					
Adult man	h	1.96	Mittal and Dhawan (1988)	8 h	300
Women	h	1.57	Mittal and Dhawan (1988)	8 h	300
Diesel	L	56.31	Mittal and Dhawan (1988)		
Farm machinery	h	62.7	Kitani (1999)	H	1,400
Organic manure	t ha^−1^	0.3	Kitani (1999)	T ha^-1^	300
Chemical fertilizers					
N	kg	60.6	Kitani (1999)	kg	6
P_2_O_5_	kg	11.1	Kitani (1999)	kg	24
K_2_O	kg	6.7	Kitani (1999)	kg	16
Irrigation water	m^3^	1.02	Singh et al. (2019)	per irrigation	100
Pesticides	kg	120	Kitani (1999)	lit	112
Rice seed	kg	14.7	Kitani (1999)	kg	40
Outputs					
Rice	kg	14.7	Mandal et al. (2015)	kg	19.4
Rice straw	kg	13.4	Mandal et al. (2015)	kg	2.5

Net energy (NE) = Output energy **−** input energy

$\text{Energy use efficiency }\left(\text{EUE}\right)=\;\frac{\text{ Output energy }\left({\text{MJ ha}}^{-1}\right)\;}{\text{input energy }\left({\text{MJ ha}}^{-1}\right)\;}$

$\text{Energy productivity }\left(\text{EP}\right)\;=\frac{\text{ Crop economic yield }\left({\text{kg ha}}^{-1}\right)\;}{\text{input energy }\left({\text{MJha}}^{-1}\right)}$

$\text{Specific energy }\left(\text{SE}\right)\;=\frac{\text{input energy }\left({\text{MJha}}^{-1}\right)\;}{Crop\;economic\;yield\;\left({\text{kg ha}}^{-1}\;\right)}$


$$\begin{array}{c} \text{Energy profitability }\left(\text{PE}\right)\\ \;=\frac{Net\;energy\;return\;\left(MJ\;ha^{-1}\right)}{input\;energy\;\left(MJ\;ha^{-1}\right)} \end{array}$$

### 2.5 Soil sampling and processing for determination of soil properties

For the laboratory analysis of soil properties, four replicated composite soil samples were collected randomly from each treatment using an augur (depth of 0–15 cm) at panicle initiation (PI) stage and after harvesting of rice, using the quartering method. For enzymatic assays, one portion of fresh soil samples was stored in the refrigerator at 4°C. The remaining portion was air dried, ground, and passed through a 2-mm sieve to prepare a final processed soil sample for analysis. Plant residues, root debris, and gravels were thoroughly removed before processing the soil sample.

### 2.6 Soil chemical and biological soil parameters

Soil chemical properties like soil pH and Eh (mV) were monitored at 3–7-day intervals throughout the seasons using pH and ORP (Star A3210 series) portable meter with a platinum-calomel electrode submerged in the reduced zone. For fractional analysis of labile carbon pool, air dried, ground, and passed through a 2-mm sieve, soil samples were taken. For the extraction of readily mineralizable carbon (RMC), 0.5 M K_2_SO_4_ (Inubushi et al., 1991) was used followed by wet digestion with dichromate (Vance et al., 1987). Chloroform fumigation extraction method described by Witt et al. (2000) was used for the analysis of microbial biomass carbon (MBC). For the analysis of other three fractions of carbon, *viz.*, permanganate oxidizable carbon (POXC), water soluble carbohydrate carbon (WSC), and soil oxidizable organic carbon, the methods described by Blair et al. (1995); Haynes and Swift (1990), and Walkley and Black (1934), respectively, were followed. The analysis of microbial population and enzymatic activities was done with field moist soil sample. Before analysis, moisture content of soil was determined on oven-dry equivalent weight basis. The soil dehydrogenase activity was determined colorimetrically using the triphenyl tetrazolium chloride (TTC) reduction technique (Casida et al., 1964). The determination of soil urease activity was done colorimetrically with diacetyl monoxim assay method described by Tabatabai (1972). On the other hand, β-glucosidase enzyme activity was determined with the help of the following method described by Dick (1994) in which colorimetric estimation of p-nitrophenol was done, which was produced by incubating soil at 37°C for 1 h with p-nitrophenol-d-glucopyranoside, modified universal buffer pH 6.0 and toluene followed by extraction with calcium chloride and tris (hydroxymethyl) amino methane. The total microbial count was estimated using serial dilution plate count method (Sikora et al., 1983), bacterial colony counts by Waksman (1927), fungal colony count by Ryckeboer et al. (2003), and Actinomycetes colony count by Williams and Davies (1965), and expressed as colony forming units (CFU) g^−1^ dry soil. Finally, soil health index was calculated taking labile carbon pool, microbial population, and soil enzyme activity.

### 2.8 Greenhouse gas flux measurement

Throughout the growth season, the flux of methane (CH_4_), carbon dioxide (CO_2_), and nitrous oxide (N_2_O) from each treatment was measured using a manual closed chamber technique. Aluminum base plate (53 cm × 37 cm × 10 cm length × width × height) with 2-cm-wide channel on the rim were inserted into the soil covering four rice plants. During the time of gas sampling, a manual closed chamber (53 cm × 37 cm × 71 cm length × width × height) was placed on the channel of the base, and channel was filled with water in order to keep the base and chamber air tight. Gas samples were collected shortly after showing/transplanting and at 3–7-day intervals during the crop growing season (depending on phenological phases of the crop) (Mohanty et al., 2018). CH_4_ (ppm), CO_2_ (ppm), and N_2_O (ppb) concentrations in the gas samples were analyzed using a gas chromatograph (M/s Thermo Scientific) using the equation as follows:

${\text{CH}}_{4}\text{flux }\left({\text{mg m}}^{-2}{\text{h}}^{-1}\right)\text{ =}\frac{XCH_{4}\times EBV\times STP\times 16\times 60\times 10^{-3}\;}{22.4\times A\times T}$


$$\begin{array}{c} {\text{N}}_{2}\text{O flux }\left({\text{µg m}}^{-2}{\text{h}}^{-1}\right)\\ \text{ =}\frac{XN_{2}O\times EBV\times STP\times 44\times 60\times 10^{-3}\;}{22.4\times A\times T} \end{array}$$

XCH_4_ = the difference in CH_4_ concentrations (ppm) at 0 min and at 30 min.

XN_2_O = the difference in N_2_O concentrations (ppb) at 0 and 30 min.

EBVSTP = effective chamber volume at standard temperature and pressure (liter).

T = time interval (minutes) between the initial (0 min) and final (30 min) sample after chamber implantation.

A = base plate area (m^2^).

Fluxes of CH_4_, N_2_O, and CO_2_ were computed for days without a sample event using sequential linear interpolation of the sample days’ mean emissions (Kumar et al., 2016). The season’s cumulative CH_4_, N_2_O, and CO_2_ emissions (kg ha^−1^) were calculated by adding daily fluxes in sampling and non-sampling days.

### 2.9 Global warming potential and greenhouse gas intensity

The global warming potential (GWP), greenhouse gas intensity (GHGI), and carbon equivalent emission (CEE) of each rice production techniques were calculated as per IPCC (2007) and protocols proposed in Bhatia et al. (2005).

$\begin{array}{c} \text{GWP }\left({\text{kg CO}}_{2}\text{eq}{\text{. ha}}^{-1}\right)\\ = 24{.5x CH}_{4}\left({\text{kg ha}}^{-1}\right){+ 320x N}_{2}\text{O }\left({\text{kg ha}}^{-1}\right)\\ {\text{ + CO}}_{2}\left({\text{kg ha}}^{-1}\right) \end{array}$

$\text{GHGI }\left({\text{kg CO}}_{2}{\text{-eq kg}}^{-1}\text{grain}\right)=\frac{GWP}{RY}$


$$\text{CEE(kg)}=\frac{GWP\times 12}{44}$$

where GWP is global warming potential in kg CO_2_ eq. ha^−1^, and RY is rice grain yield in kg.

### 2.10 Estimation of profitability of different rice establishment techniques

The economics of rice cultivation in all the rice production techniques was estimated using the following equation (Gulati et al., 2022).


$$\begin{array}{c} \text{Total cost of cultivation (CC)\$=}\\ \sum_{i=1}^{i=n}\left(Q_{i}\times P_{i}\right)\sum_{i=1}^{i=n}OC_{i} \end{array}$$

$$\text{Gross return (GR) \$=}\sum_{i=1}^{i=n}\left(Y_{r}\times P_{r}\right)$$

$$\begin{array}{c} \text{Net benefit }(\text{NB})\text{ \$ = }\\ \text{Gross Return }\left(\text{GR}\right)\text{ - Total Cost of Cultivation }\\ \left(\text{CC}\right) \end{array}$$

$$\begin{array}{c} \text{Benefit: cost ratio (B:C Ratio)}=\\ \frac{Gross\;return}{Total\;cost\;of\;cultivation\;} \end{array}$$

$$\text{Production efficiency (PE)=}\frac{Grain\;yield}{crop\;duration\;}$$

$$\text{Monetary efficiency (ME)=}\frac{yield}{Net\;Benefit\;}$$

where Q_i_ is the quantity of agricultural input used, P_i_ is the market price of agricultural input, OC_i_ is the agricultural operational cost, Y_R_ is the yield of rice grain and straw yield (Mg ha^-1^), and P_r_ is the minimum support price (MSP) (CACP, 2021) for rice grain and market price for straw. After calculating the cost of cultivation, gross return, and net profit, these figures are converted from Indian rupees to US dollars by dividing by 73.5.

### 2.11 Drivers of yield and greenhouse gas emissions

A forward stepwise regression was used to establish a relationship between crop yield and greenhouse gas emissions with different soil properties and to screen the important soil properties governing the yield and GHG emission variability. For this, at first, two intercept models were fitted, i.e., rice yield ~ intercept and GHG emission ~ intercept. Furthermore, a full model was developed, i.e., yield and emission dependent on all the soil properties. Finally, the step function of R was used to create a stepwise model in R to screen the important variables.

### 2.12 Estimation of climate smartness index

A modified climate smartness index (CSI) for different rice production techniques was calculated based on the procedure proposed in Arenas-Calle et al. (2019). For the calculation of CSI, a set of indicators were selected to represent different indicators of sustainability (productivity, Irrigation water productivity, energy productivity, nitrogen partial factor productivity (PFPN), global warming potential, and economy). The indicators were normalized using the minimum (min) and maximum (max) normalization (N) approach (OECD, 2008; Mazziotta and Pareto, 2013). This normalization rescaled the CSI indicator values from 0 to 1. Furthermore, either the “more is better” or “less is better” technique was used depending on whether the indicator has a beneficial or cost criteria, to ease the comprehension of each indicator’s contribution in the CSI (Pollesch and Dale, 2016). For this type of normalization, min and max thresholds of productivity, nitrogen partial factor productivity (PFPN), water productivity (WP), energy productivity (EP), global warming potential (GWP), and benefit–cost ratio (B:C ratio) data for rice production system were used. The maximum and minimum threshold values of different rice production techniques were derived from different published articles of the same location. Finally, the normalized indicators were weighted. In this regard, three approaches were used for weighting these indicators: (1) expert opinion (Brooks et al., 2005); (2) by giving equally weight to each indicator (Gan et al., 2017); and (3) Statistical approach by principal components analysis (PCA) (Jiang et al., 2020). Here, we used PCA to assign significant weight to each CSA indicators so that different indicators affect CSI unequally. The following equations were used for normalization and weightage computation.

$\text{N= }\frac{\left(n_{obs}\right)}{\left(n_{max}\right)}$ more is better

$\text{N= }\frac{\left(n_{obs}\right)}{\left(n_{min}\right)}$ less is better


$$\text{W= }\frac{ev_{i}^{2}}{\sum_{I=1}^{I=n}ev_{i}^{2}}$$

$$\text{CSI=}\overset{n}{\underset{i=1}{\;\sum }}W_{i}N_{i}$$

where CSI= climate smartness index, W= weightage of each indicator, ev = Eigen vector from PCA analysis, N= normalized value, P=productivity (min= 1.97 t ha^−1^, max= 6.55 t ha^−1^), WP (min= 0.1 kg grain m^−3^, max = 70.1 kg kg^−1^), (PFPN (min = 25 kg kg^−1^, max 70.1 kg kg^−1^) EP (min = 0.05 max= 0.3), GWP (min = 1,970 kg CO_2_-eq/ha, max= 5,109 kg CO_2_-eq/ha), and B:C ratio (min=1.15, max = 2.2) are the minimum and maximum value these indicators collected from systematic review.

### 2.12 Statistical analysis

The data for different indicators were analyzed using the SPSS version 21.0 software. The data for various indicators were analyzed using the one-way analysis of variance (ANOVA). If the ANOVA was found significant at the 0.05 level of probability, Duncan’s multiple range test (DMRT) was performed to differentiate the effect of treatment means (Gomez and Gomez, 1984).

## 3 Result

### 3.1 Crop yield and water and nitrogen use efficiency

The rice production techniques had significant effect on rice grain yield, biomass yield, and harvest index (**Table 3**). Rice grown under SRI-AWD method produced 5.04%–39.4% and 4.73%–40.1% higher grain yield than other production techniques in the winter season of 2020 and 2021, respectively. The DSR-based AWD and CF rice production technique had 15.6% and 22.5% higher grain yield than farmer practices (FPR-CF); however, the rice yield under AWD and CF under DSR was similar. In addition, there was no significant difference observed in yield of DSR-CF and TPR-AWD during both the season. TPR methods had 21.9% higher yield in AWD and 33.3% in CF compared to farmer practices (**Table 3**). The straw yield was largest in the SRI-AWD treatment followed by TPR-CF> DSR-CF>TPR-AWD>DSR-AWD > FPR-CF, while the harvest index was highest in the SRI-AWD and lowest in FPR-CF among different rice production techniques (**Table 3**).

**Table 3:** Yield, irrigation water productivity, and nitrogen partial factor productivity of rice crop as influenced by different rice production techniques.

Treatment	Grain yield (Mg ha^−1^)		Straw yield (Mg ha^−1^)		Harvest index (%)		Irrigation water productivity (kg m^−3^)		Nitrogen partial factor productivity (%)	
	Rabi 2020	Rabi 2021	Rabi 2020	Rabi 2021	Rabi 2020	Rabi 2021	Rabi 2020	Rabi 2021	Rabi 2020	Rabi 2021
SRI-AWD	5.41a	5.53a	6.67a	6.79a	0.45a	0.45a	0.53a	0.54a	63.7a	65. 0a
DSR-CF	4.74c	4.83c	6.55b	6.54c	0.43c	0.42c	0.31d	0.31d	47.3c	48.3c
DSR-AWD	4.52d	4.65d	6.14e	6.34c	0.42cd	0.42c	0.42b	0.44b	45.2d	46.5d
TPR-CF	5.15b	5.28b	6.55b	6.65b	0.44b	0.44b	0.34c	0.35c	51.4b	52.8b
TPR-AWD	4.72c	4.82c	6.46c	6.56b	0.42d	0.42c	0.43b	0.44b	47.2c	48.2c
FPR-CF	3.88e	3.95e	6.07f	6.24c	0.39e	0.39d	0.24e	0.24e	38.8e	39.5e
SEm (±)	0.03	0.04	0.02	0.05	0.003	0.002	0.01	0.01	0.30	0.36
LSD ≤ 5%	0.06	0.07	0.11	0.12	0.01	0.003	0.02	0.02	0.64	0.76

SRI-AWD -T_1_; DSR-CF -T_2_; DSR-AWD -T_3_; TPR-CF -T_4_; TRP-AWD -T_5_; FPR-CF-T_6_ (different letters for each parameter show significant difference at p ≤ 0.05 by Duncan’s multiple range test).

Total water use was higher in conventional method of rice cultivation (FPR-CF), followed by TPR-CF, DSR-CF, TPR-AWD, SRI-AWD, and DSR-AWD, respectively (**Table 3**). The water saving was largest under SRI-AWD (37.5%), followed by DSR-AWD (34.7%) and TPR-AWD (33.5%) as compared to FPR-CF. Significantly largest grain yield per unit quantity of water used (water productivity) was found under SRI-AWD (0.53, 0.54 kg m^−3^) and least in FPR-CF (0.24 and 0.24 kg m^−3^) during both the seasons. Similarly, the water productivity for DSR-AWD was observed 0.42 and 0.44 kg m^−3^, whereas the water productivity under DSR-CF was 0.31 and 0.31 kg m^−3^ during both the crop growing season. Among all the rice production techniques, on an average, the AWD technique increased the water productivity by 78.3% compared to CF water management (**Table 3**). Nitrogen partial factor productivity (PFPN) was significantly varied among the rice production techniques (**Table 3**). It varied from 38.8 to 65.0 kg grain per kg N applied, whereas the highest was observed in SRI-AWD and least in FPR-CF. The DSR- CF had on an average 4.25% higher PFPN as compared to DSR-AWD, whereas the TPR-CF had 8.4% higher NUE as compared to TPR-AWD. The PFPNs under FPR-CF were 38.8 and 39.5 kg grain per kg N.

### 3.2 Energy budgeting

The input energy requirement for rice production varied under different rice production techniques (**Table 4**). The total energy requirement was largest in TPR-CF, which was nearly similar to the energy requirement under farmer’s practices (1.74% higher over farmer practices). The least total energy requirement was recoded in DSR-AWD, which was on an average 26.1% lesser than FPR in both seasons (**Table 4**). DSR production technique required 23.8% less energy as compared to conventional rice cultivation, with the majority of the energy savings obtained from diesel, machinery, and labor. The energy output was found largest in SRI-AWD, which was 19.8% more than the FPR-CF (**Table 4**). All the indices of energy use efficiency, i.e., EUE, net energy, energy productivity, and profitability, differed significantly among the rice production practices (**Table 4**). The largest net energy was observed in SRI-AWD technique, which was on an average 28% higher than FPR-CF during both the year. The largest energy use efficiency was obtained under DRS-AWD method of rice production, which was 45.7% larger than FPR-CF technique. The different energy-efficient rice production practices improved the EUE by 27.3%–45.4% and 27.3–46.1% during 2020 and 2021, respectively.

**Table 4:** Energy input–output relationship of rice crop as influenced by different rice production techniques.

Treatment	Input energy (GJ ha^−1^)	Output energy (GJ ha^−1^)	Net energy (GJ Ha^−1^)	Energy use efficiency	Energy productivity (kg MJ^−1^)	Specific energy (MJ kg^−1^)	Energy profitability (MJ ha^−1^)
	Rabi 2020							
SRI-AWD	23.8d	174a	150a	7.30b	0.23a	1.96e	6.30b
DSR-CF	22.5e	159c	136c	7.07c	0.21b	2.02d	6.07c
DSR-AWD	21.2f	156d	135c	7.36a	0.21b	1.95e	6.36a
TPR-CF	29.2a	168b	139b	5.80e	0.18d	2.46b	4.80e
TPR-AWD	24.9c	160c	135c	6.44d	0.19c	2.21c	5.44d
FPR-CF	28.7b	146e	117d	5.06f	0.13e	2.84a	4.06f
SEm (±)	0.02	0.83	0.86	0.03	0.001	0.01	0.03
LSD ≤ 5%	0.06	1.80	1.74	0.07	0.002	0.02	0.07
	Rabi 2021							
SRI-AWD	23.8d	176a	152a	7.36b	0.23a	1.94e	6.36b
DSR-CF	22.5e	160cd	138c	7.13c	0.21b	2.00d	6.13c
DSR-AWD	21.2f	158d	137c	7.45a	0.22b	1.93e	6.45a
TPR-CF	29.2a	172b	143b	5.94e	0.19d	2.40b	4.94e
TPR-AWD	24.9c	162c	137c	6.49d	0.19d	2.19c	5.49d
FPR-CF	28.7b	146e	118d	5.10f	0.14e	2.82a	4.10f
SEm (±)	0.02	0.92	0.95	0.04	0.001	0.01	0.04
LSD ≤ 5%	0.06	1.96	1.97	0.08	0.002	0.02	0.08

SRI-AWD -T_1_; DSR-CF -T_2_; DSR-AWD -T_3_; TPR-CF -T_4_; TRP-AWD -T_5_; FPR-CF-T_6_ (different letters for each parameter show significant difference at p ≤ 0.05 by Duncan’s multiple range test).

### 3.3 Soil redox potential, greenhouse gas emission and global warming potential

**Figure 1:**
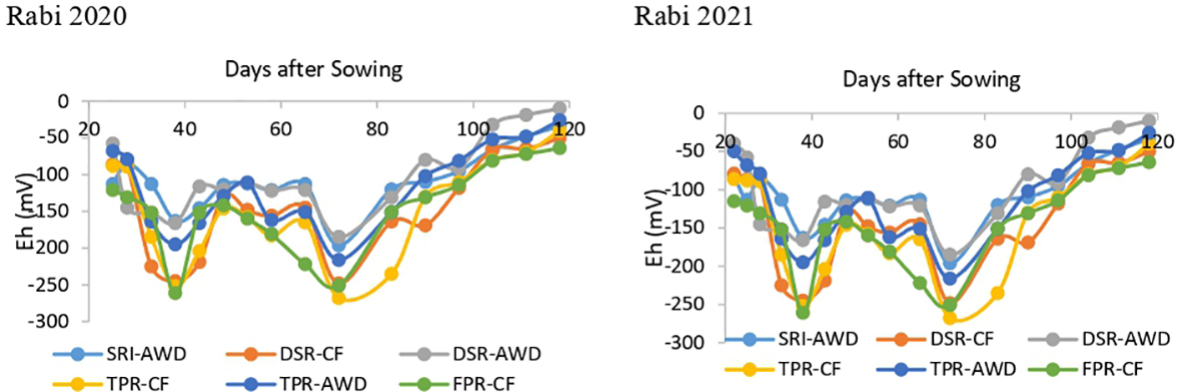
Effect of different rice production techniques on soil Eh (redox potential) in rice crop. SRI-AWD-T1; DSR-CF-T2; DSR-AWD-T3 TPR-CF-T4; TPR-AWD-T5; FPR-CF-T6.

Soil pH and Eh value during rice growing period varied in between 5.5–7.1 and 20 to −261 mV among different rice production techniques during both years. After transplanting/sowing under continues flooding conditions, soil Eh declined until panicle initiation stage, then gradually increased in all CF treatments (**Figure 1**). The lowest Eh value was recorded under DSR-AWD, TPR-AWD, and SRI-AWD treatment, which was significantly lower than other CF treatments. The average seasonal redox potential under AWD based techniques was 26.2% more than the CF treatments.

**Figure 2:**
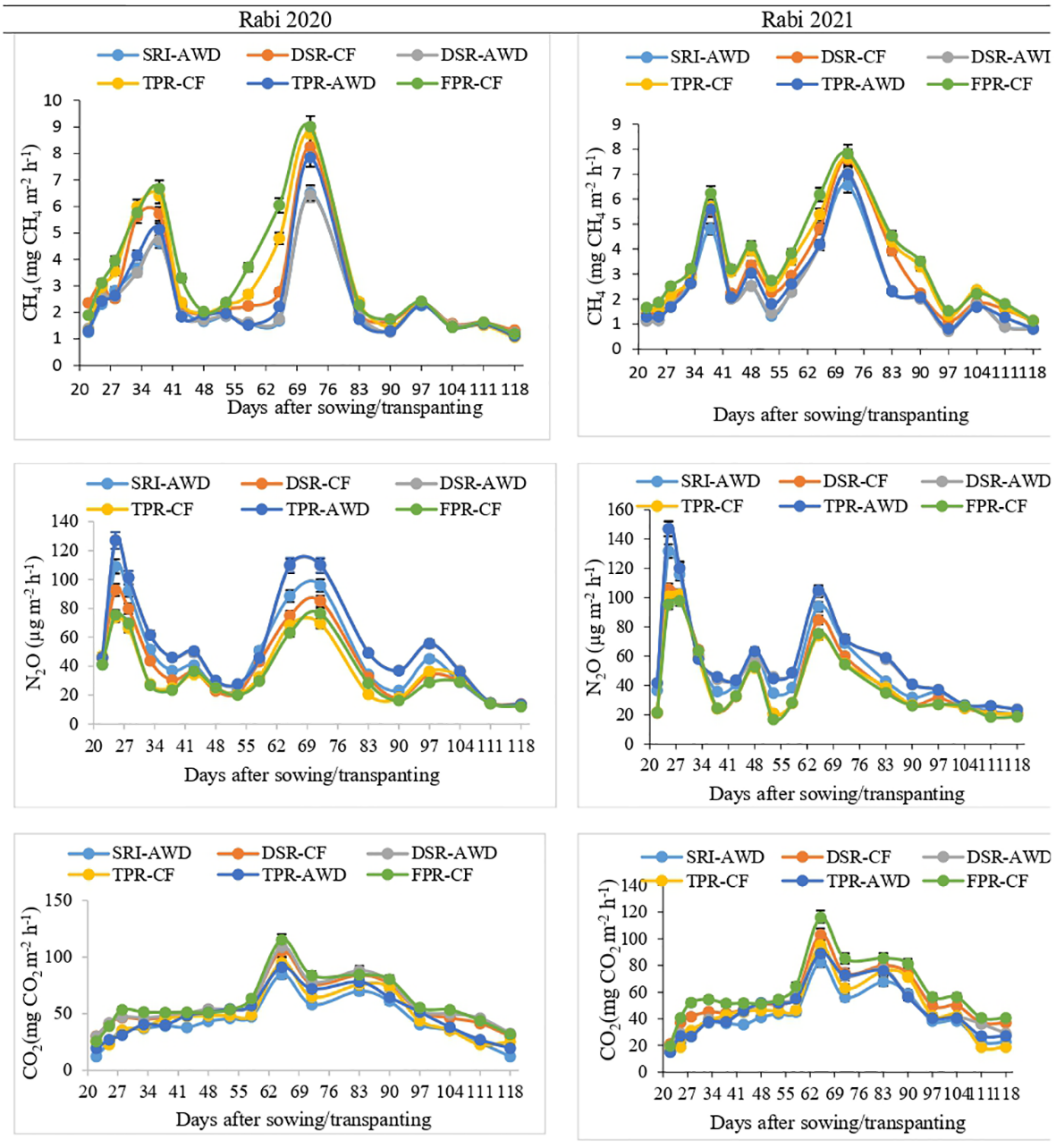
Methane (CH_4_), nitrous oxide (N_2_O), and carbon dioxide (CO_2_) flux from soils during different rice production technique of rice crop.

Methane flux under different rice production techniques varied significantly (*p*< 0.05) in between 1.06–9.01 mg m^−2^ h^−1^ and 0.72–7.83 mg m^−2^ h^−1^, during the rice production season of 2020 and 2021, respectively (**Figure 2**). The highest methane flux (9.201 and 7.83 mg m^−2^ h^−1^) was recorded during panicle initiation stage (at 72th Julian day of 2020 and 2021) under different rice production technique during both the season (**Figure 2**). The cumulative CH_4_ emission under FPR-CF was significantly (*p*< 0.05) higher than that under SRI-AWD and DSR-AWD during both years (**Table 5**). Alike methane emission, the N_2_O emissions varied significantly under different rice establishment techniques. The largest N_2_O emission (127.0 and 147 μg m^2^ h^−1^) was observed under the TPR-AWD treatments, which was at per with DSR-AWD (126.8 and 147 μg m^−2^ h^−1^), whereas TPR-CF had the least nitrous oxide emission (126.8 and 147g m^2^ h^−1^) in both years (**Figure 2**). The cumulative N_2_O emission was significantly (*p*< 0.05) higher in AWD treatments (DSR-AWD, TPR-AWD, and SRI-AWD), which was 27.9% to 42.3% higher compared to CF treatments (DSR-CF, TPR-CF, and FPR-CF) during both years (**Table 5**). Significant effects of different rice production techniques on CO_2_ emission from rice field were observed (**Figure 2** and **Table 5**). The CO_2_ fluxes varied from 12.3 to 115.2 mg CO_2_ m^−2^ h^−1^ in the rice production system during both years. Regardless of the treatments, the CO_2_ flux increased till flowering and decreased thereafter. The average cumulative CO_2_ emissions under different rice production techniques ranged between 1,146–1,647 kg ha^−1^ and 1,147 to 1,625 kg ha^−1^ in 2020 and 2021, respectively (**Figure 2** and **Table 5**).

**Table 5:** Greenhouse gas emissions, GWP, and carbon equivalent emission in different rice production techniques.

Treatment	CH_4_ emission (kg ha^−1^)		CO_2_ emission (kg ha^−1^)		N_2_O emission (kg ha^−1^)		GWP of rice system (kg CO_2_ ha^−1^)		Carbon equivalent emission (CEE) (kg C ha^−1^)	
	Rabi 2020	Rabi 2021	Rabi 2020	Rabi 2021	Rabi 2020	Rabi 2021	Rabi 2020	Rabi 2021	Rabi 2020	Winter 2021
SRI-AWD	62.6e	63.6f	1,179e	1,146f	1.22b	1.33ab	3106e	3,131d	847e	854d
DSR-CF	76.9c	78.7c	1,527c	1,474b	1.07bc	1.13c	3757b	3,766b	1,024b	1,027b
DSR-AWD	63.2e	65.6e	1,310d	1,323c	1.46a	1.47a	3326d	3,401c	980c	928c
TPR-CF	84.4b	88.2b	1,576b	1,265e	0.94d	1.09b	3680b	3,773b	1,004b	1,029b
TPR-AWD	66.9d	68.2d	1,303d	1,287d	1.46a	1.48a	3412c	3,433c	931d	937c
FPR-CF	91.2a	94.6a	1,625a	1,647a	0.92d	1.11b	4169a	4,319a	1,137a	1,178a
SEm (±)	1.20	0.65	15.0	3.86	0.06	0.11	35.1	31.8	9.57	8.69
LSD ≤ 5%	2.94	1.45	33.5	8.54	0.11	0.25	78.2	71.0	21.3	19.4

SRI-AWD-T_1_; DSR-CF-T_2_; DSR-AWD-T_3_; TPR-CF-T_4_; TRP-AWD-T_5_; FPR-CF-T_6_ (different letters for each parameter show significant difference at p ≤ 0.05 by Duncan’s multiple range test).

The methane, nitrous oxide, and carbon dioxide emission were converted to GWP based on CO_2_ equivalent values of respective GHGs. The GWP under different rice production techniques varied significantly in both years. The largest GWP was observed in FPR-CF, which was 9.9%–25.5% and 12.8%–27.5% higher than other rice production techniques during 2020 and 2021, respectively. The AWD irrigation with SRI establishment techniques had least (3,106–3,131kg CO_2_ ha^-1^) GWP, which was 25.5% and 27.5% lesser than the GWP under FPR-CF during both seasons (**Table 5**). The DSR-AWD and TPR-AWD had 20.2%–21.3% and 18.2%–20.5% lesser GWP than FPR-CF during both the seasons.

### 3.4 Soil health indicators

**Figure 3:**
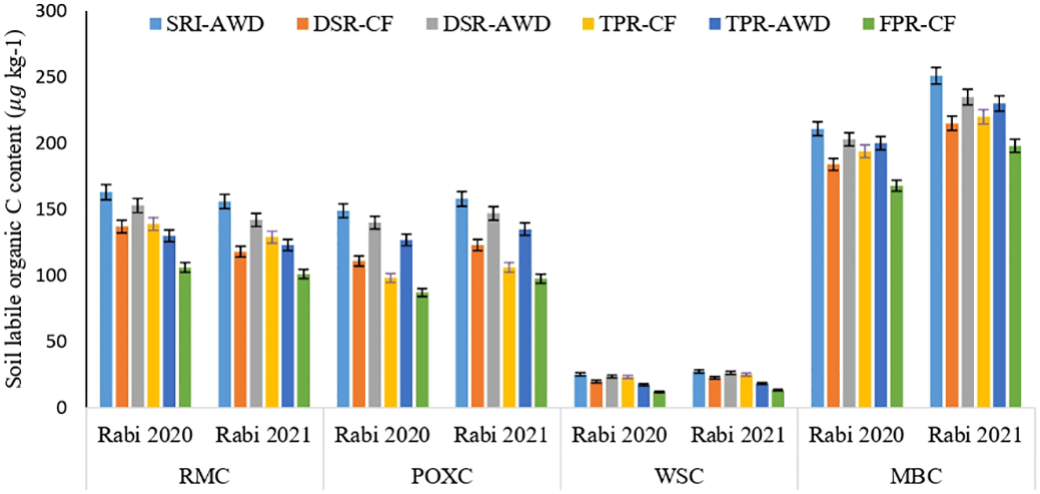
Soil labile C content such as readily mineralizable carbon (RMC), permanganate oxidizable carbon (POXC), water soluble carbohydrate carbon (WSC), and microbial biomass carbon (MBC) (g kg^−1^) in Rabi 2020 and Rabi 2021 under different rice production techniques in rice–rice cropping system.

**Figure 4:**
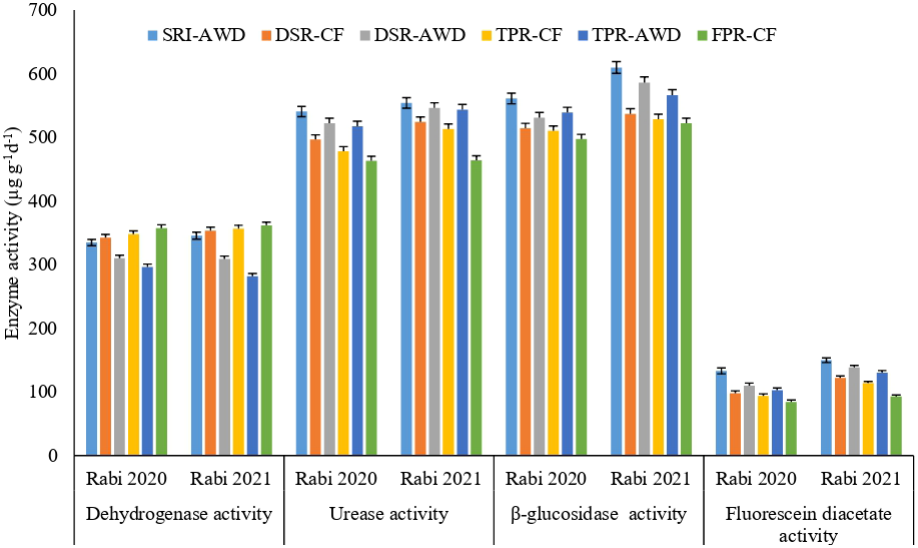
Soil dehydrogenase activity, urease activity, β−glucosidase activity and fluorescein diacetate activity (μg TPF g^−1^ day^−1^) during Rabi 2020 and Rabi 2021 under different rice production techniques in rice crop.

The soil health indicators, i.e., the labile carbon fractions, soil enzymes, and microbial parameters at panicle initiation state of rice soil, varied significantly across different rice production techniques (**Figure 3**, **4** and **Table 6**). In addition, the rice production practices significantly affected different C-fractions (RMC, WSC, and POXC) (**Figures 3**, **4** and **Table 6**). The mean RMC, POXC, and WSC contents were significantly higher in SRI-AWD, which were 1.54, 1.58, and 2.05 times higher than the FPR-CF treatment. The RMC concentration was significantly increased in SRI-AWD practices by 156 and 163 μg kg^−1^ during 2020 and 2021, respectively, than FPR-CF. Soil POXC concentration was greater in DSR-AWD and TPR-AWD, as compared to FPR-CF. MBC of soil remained unaffected by different rice production techniques, and the largest MBC was observed under SRI-AWD (211–251 μg kg^−1^ soil), while the least MBC was observed under FPR-CF (168–198 μg kg^−1^ soil). The microbial populations of bacteria, fungi, actinomycetes, heterotrophs, denitrifiers, and methanogen recorded at PI stages varied significantly across different rice production techniques (**Table 6**). The largest microbial population was observed in the SRI-AWD method of rice cultivation, which was 20%–45% higher as compared to FPR-CF. The trend in the build-up of bacteria, fungi, and Actinomycetes populations was largest under SRI-AWD, followed by TPR-AWD, and least in FPR-CF. Similarly, the heterotrophs and methanogens population were higher in FPR-CF, followed by TPR-CF, and least in SRI-AWD, whereas the denitrifiers population was higher in DSR-AWD and TPR-AWD in both the season. The soil enzymes activities, like DHA, urease, β-glucosidase, and FDA, were significantly improved with different rice production practices (**Figure 4**). Among the different rice production techniques, the SRI-AWD practices had largest enzymatic activities like urease, β-glucosidase, and FDA, whereas the FPR-CF had least enzymatic activities, which were at par with DSR-CF and TPR-CF. The urease, β-glucosidase, and FDA activities increased by 19.4%, 16.7%, and 61.2%, respectively, in SRI-AWD, as compared to FPR-CF during 2021 and 16.7%, 12.1%, and 57.3% during 2020, respectively. The maximum DHA activity was found in FPR-CF (362 and 357 g kg^−1^) and least in TPR-AWD (296.5 and 282 g kg^−1^).

**Table 6:** Soil microbial population dynamics of rice crop influenced by different rice production techniques.

Treatment	Bacterial population (CFU × 10^5^ g^−1^ dry soil)		Fungus (CFU × 10^3^ g^−1^ dry soil)		Actinomycetes (CFU × 10^4^ g^−1^ dry soil)		Heterotrops (CFU × 10^6^ g^−1^ dry soil)		Denitrifiers (CFU x 10^5^ g^−1^ dry soil)		Methanogens (log MPN g^−1^ dry soil	
	Rabi 2020	Rabi 2021	Rabi 2020	Rabi 2021	Rabi 2020	Rabi 2021	Rabi 2020	Rabi 2021	Rabi 2020	Rabi 2021	Rabi 2020	Rabi 2021
SRI-AWD	12.8a	17.8a	2.46a	3.72a	4.23a	5.98a	2.1b	2.5cd	5.4ab	6.3ab	3.3bc	4.3b
DSR-CF	10.9b	13.2b	1.78ab	2.78b	3.05d	4.0b	2.3b	2.9b	5.2ab	6.1ab	3.6ab	4.5bc
DSR-AWD	10.1ab	12.7b	1.74ab	2.49b	3.45c	4.45b	2.2b	2.2de	5.5a	6.4a	3.4abc	4.4bc
TPR-CF	11.5ab	13.4b	1.86ab	2.86b	3.22cd	3.98b	2.3b	2.8bc	5.1b	6.1ab	3.6ab	4.7ab
TPR-AWD	11.2ab	11.3b	1.74ab	2.56b	3.73b	4.3b	2.1b	2.1e	5.5a	6.4a	3.2c	4.1b
FPR-CF	10.1b	12.1b	1.66c	1.45c	3.23cd	3.23c	2.4a	3.5a	5.1b	5.8b	3.7a	4.8a
SEm (±)	0.71	0.70	0.05	0.15	0.08	0.19	0.08	0.11	0.10	0.15	0.12	0.10
LSD ≤ 5%	0.21	2.07	0.14	0.44	0.24	0.55	0.24	0.33	0.30	0.45	0.35	0.30

SRI-AWD-T_1_; DSR-CF-T_2_; DSR-AWD-T_3_; TPR-CF-T_4_; TRP-AWD-T_5_; FPR-CF-T_6_ (different letters for each parameter show significant difference at p ≤ 0.05 by Duncan’s multiple range test).

### 3.5 Drivers of crop yield and greenhouse gas emission

The stepwise regression was used to screen the important soil properties governing the yield and emission variability. WSC and bacterial population (BP) positively affected the rice yield, whereas the methanogenesis activity and POXC negatively affected the rice yield (although very small negative effect of permanganate oxidizable carbon). A unit change in WSC was associated with 0.06 t increase in rice yield, whereas the unit increase in methanogenesis was associated with 0.33 t ha decrease in rice yield when all other factors are present at their mean level (**Table 7**). As expected, the denitrifiers and methanogenesis population positively affected the GHG emission from the rice field, i.e., a unit increase in methanogens and denitrifiers population was associated with 208 and 189 CO_2_-eq increase in GWP when all other factors are present at their mean level. The bacterial population, urease activity, and readily mineralizable carbon negatively affected the GWP (**Table 7**).

**Table 7:** Stepwise regression between yield and GWP with soil properties.

Parameters	Yield	Emission
(Intercept)	3.447308***	7,124.55***
WSC	0.064905***	−2.9401
Bacterial population	0.049683*	−33.2759**
Methanogenesis	−0.33959***	208.7053***
POC	−0.00508*	0.612
Heterotroph	0.223876	113.0558
MBC	0.00367	−1.4006
UA		−5.1213***
BGTA		−3.188*
Denitrifiers		189.1699**
RMC		−4.9061*

*(p< 0.05); **(p< 0.01), ***(p< 0.001) in parameters coefficients indicate level of significance in the stepwise regression analysis.

### 3.6 Profitability of different rice production techniques

Production cost for various rice production techniques varied significantly across different crop production techniques (**Table 7**). The cost of production was largest in TPR-CF, followed by TPR-AWD, which was at par with FPR-CF and least in DSR-AWD. The cost of production under FPR-CF was 1.12%–4.62% higher than other rice production techniques, i.e., SRI AWD, DSR-AWD, and DSR-CF during both years. Gross and net return and benefit–cost ratio varied significantly across different rice production techniques (**Table 7**). SRI-AWD method fetched significantly largest gross and net return over other rice production techniques. The gross and net return under SRI-AWD were 34.9% and 122%, respectively, higher than FPR-CF in both years. The largest B:C ratio was observed in SRI-AWD and least in FPR-CF in both years. Among different rice production techniques, the production efficiency (PE) varied from 25.8% to 36.2% and 31.0% to 36.9% in 2020 and 2021, respectively, whereas the monetary efficiency (ME) varied from 9.65% to 15.2% and 9. 32% to 14.7%. The maximum PE was observed in SRI-AWD, whereas the least PE was observed under FPR-CF. The SRI-AWD techniques were most profitable than all other rice production practices.

### 3.7 Climate smartness index

**Figure 5:**
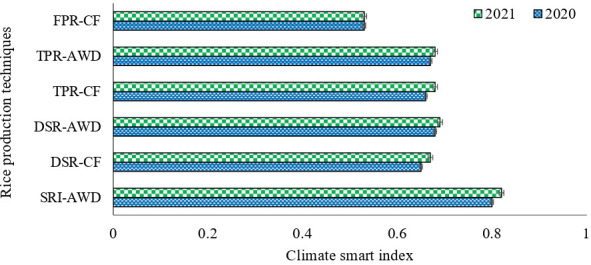
Climate smartness index (CSI) during both the season under different rice production techniques in rice crop.

The multiple indicators were evaluated using a composite index, i.e., climate smart index, to screen the production practices that are sustainable over long run. The CSI varied between −1 to +1. The composite CSI varied significantly across different rice production methods (**Figure 5**). The SRI-AWD had the largest CSI, i.e., 0.80 and 0.82, which indicates that CSI had higher grain yield, higher water productivity, higher energy efficiency, higher profitability, and least GHG emission, whereas the least CSI score was observed under FPR-CF, i.e., 0.53 during both years, which indicates the least yield, energy efficiency, and largest emission under FPR-CF. The CSI under CSI-AWD was 1.50 and 1.55 times greater than the farmer’s practices. The performance of the DSR-AWD and TPR-AWD was in between that of SRI-AWD and FPR-CF. The CSI score under DSR-AWD and TPR-AWD techniques of rice production was 4.6% and 1.54% higher than DSR-CF and TPR-CF, respectively.

## 4 Discussion

### 4.1 Effect of different rice production techniques on productivity, profitability, and energy use efficiency.

The congenial growth conditions prevailed because AWD under SRI method allowed the rice plant to have a greater number of phyllochrons, increased number of tillers, and highly developed roots, before the flowering phase (Deb, 2020) and produced highest grain yield over all the rice production techniques evaluated. Our findings were in accordance with the findings of Chapagain and Yamaji (2010); Mishra and Salokhe (2011), and Nazir et al. (2022). Talla and Jena (2014) and Kumar et al. (2016) have also reported approximately 7%–30% higher grain yield in SRI-AWD over the conventional method. Lal et al. (2016) reported that younger seedlings with higher tillering ability, optimum plant population, efficient nutrient, and moisture utilization enabled SRI to produce higher grain yield. Higher grain yield in DSR was due to early seeding vigor, avoidance of transplanting shock, increased panicle number, higher test weight, and reduced poor grain sterility percentage (Gangwar et al., 2008). Compared to continuous flooding, AWD considerably reduced water use while maintaining or even increasing yields (Mishra and Salokhe, 2011; Thakur et al., 2014; Malyan et al., 2016). Although the mechanisms underlying these adaptations are unknown, AWD was found to be strongly associated with root development and access to water and nutrients at a deeper level in the soil profile (Thakur et al., 2014). AWD-induced rhizosphere drying also improved plant hormone signaling and increased grain filling rate, especially in inferior spikelet (Davies et al., 2011; Zhang et al., 2012). Zhang et al. (2009) reported an increase in rice yield by 11% due to AWD compared to continuous flooding. Moreover, yield decreased in DSR, which was also reported by recent studies (Farooq et al., 2007; Farooq et al., 2009). The yield and yield attributes are often higher in TPR because of proper utilization of available resources and efficient partitioning of photosynthates in comparison to the conventional method (Kumar et al., 2016). However, yield attributes were lower in TPR compared to that in SRI and higher over DSR in sandy clay loam soil, since the older seedlings took relatively longer time to mature. In line transplanted rice, the flowering was delayed by 12–16 days compared to DSR, which might be ascribed to transplanting shock (Joshi et al., 2013). Soil texture plays an important role in water management in rice production. Similarly, rice yield was reduced under AWD management in clay texture soil compared to loam soil because of dryness in clay soil at −20kPa compared to loam (Carrijo et al., 2017). Under the conventional system, cultural practices of transplanting of older seedlings at three to four seedlings/hill, continuous flooding of the field throughout crop would have resulted in poor root growth with quicker root degeneration (Chakraborty et al., 2017). Nirmala et al. (2021) reported that conventional method of rice production yield was reduced up to 55% compared to SRI methods. Thus, adaptation of rice production technique like SRI, DSR, and TPR (mechanical) with AWD in rice ecology may enhance overall productivity along with resilience to climate change (Lal et al., 2020; Kumar et al., 2022).

SRI-AWD rice production technique required less water and have higher grain yield, whereas DSR-AWD and TPR-AWD methods of rice production required less water use but had less yield compared to SRI-AWD. This might be attributed to less irrigation water and chemical fertilizer lowering the cost of cultivation without affecting grain yield. Increased water productivity in AWD systems could be attributed to reduced seepage and percolation loss compared to flood irrigation. However, such losses are highly reliant on a soil’s hydrological characteristics (Sharma et al., 2002; Pérez-Sánchez et al., 2012). Saving of water in AWD, SRI-AWD (20%–50%), and DSR (13%–60%) as compared to flooding was also reported by several workers (Singh et al., 2013; Dass et al., 2016; Carrijo et al., 2017; Mboyerwa et al., 2021). In SRI-AWD rice production technique, 1 kg of N fertilizer produced 65 kg of grain on average; however, when 1 kg of N fertilizer was applied to rice using the FPR-CF technique, only 38.8 kg of grain was produced. Higher N use efficiency in SRI-AWD treatment might be due to higher root growth, photosynthetic rate, and N uptake. In AWD practices, there were different losses of N, *viz*., leaching and denitrification by draining less water and leaving less stagnant water in the field. Contrarily, hypoxic conditions in continuous flooding with faulty N management in farmer’s practice inhibit root growth and N uptake. Thakur et al. (2013) reported the same, as SRI-AWD had higher PFPN than FPR-CF.

Energy was consumed at every stage of the rice production, from tillage operation to threshing. Total energy requirement for optimum production of a crop is the function of input used (seed rate, manure and fertilizer rate, herbicide and pesticide rate, irrigation, etc.), crop production method, duration of cultivar, and cropping system (Lal et al., 2020). The total energy requirement was highest in TPR-CF. It might be due to mechanical transplanter, which consumed higher energy than manual transplantation. However, FPR-CF had demanded more human labor, fertilizer, seed, and area to get more seedling to transplanting per unit area than other rice production techniques. Fertilizers used the majority of total input energy consumption, accounting for 40%–70% of total input energy compared to other input requirement, which was supported by recent findings (Singh et al., 2019; Lal et al., 2020). The second important factor of energy consumption was land preparation, which includes tillage operation, irrigation for seedbed preparation, and machinery. It was similar to the finding of Eskandari and Attar (2015). SRI-AWD had higher output energy, which could be attributed to higher yield than the other tested techniques (Lal et al., 2020). Highest EUE in DSR technique might be due to high resource use efficiency (radiation use efficiency, water productivity, and nutrient use efficiency) (Lal et al., 2020; Htwe et al., 2021). DSR technique needed less energy consumption than transplanting rice production technique, resulting in a greater EUE (Eskandari and Attar, 2015). Here, higher specific energy in FPR-CF technique indicated that more energy input is needed to produce one-unit rice grain. The B:C ratio of SRI-AWD was in accordance with findings of Singh et al. (2013) and Soni and Soe (2016). DSR-AWD was a cost-effective alternative to DSR-CF, TPR-CF, and TPR-AWD with a similar B:C ratio. Gautam et al. (2021) reported less gross return in DSR-AWD compared to transplanted rice. The least benefit–cost ratio for FPR-CF was due to higher seed rate, higher disease and weed infestation, lower grain yield, and knowledge gap in rice production (Devkota et al., 2022).

### 4.2 Effect of different rice production techniques on greenhouse gas emission and GWP

Methane gas emissions included production, oxidation, and transportation systems (Brye et al., 2016). Alternate wetting and drying (AWD) increased irrigation water efficacy in mitigating 45%–90% CH_4_ emissions compared to continuous flooding (Nalley et al., 2015; Xu et al., 2015; Liang et al., 2016). During dry spells in AWD, the reduction in CH_4_ emission is the consequence of a subtle decrease in methanogenic and methanotrophic activity along with an increased supply of oxygen (Tirol-Padre et al., 2018; Islam et al., 2020). Continuous flooding maintained anoxic soil environment (redox potential<−150 mV), which facilitated anaerobic organic matter decomposition and increased CH_4_ emissions (Minamikawa et al., 2006; Tirol-Padre et al., 2018; Islam et al., 2020). DSR reduced GHG emission by 82%–87% than TPR (Gupta et al., 2016; Mishra et al., 2021). DSR provided non-puddled soil condition, higher water percolation rate, and increased soil macro-porosity and soil pore continuity (Singh et al., 2020), thus improved gas diffusivity and increased CH_4_ oxidation. However, in TPR-CF, puddled condition restricted percolation of water and anaerobic condition was formed, which was favorable for CH_4_ emission (Harada et al., 2007; Kumar et al., 2019). Anoxic conditions were produced in FPR-CF, allowing methanogens to produce CH_4_ by anaerobic decomposition of organic materials (Bhattacharyya et al., 2016; Swain et al., 2016, Swain et al.,2018). In the AWD water regime treatment, N_2_O emissions were greater than continuous flooding plots, which varied from 0.88 and 1.5 kg N_2_O ha^−1^ worldwide (Romasanta et al., 2017; Islam et al., 2020). Previous studies have also reported the similar findings of higher N_2_O emissions in AWD (Miniotti et al., 2016; Tariq et al., 2017; Islam et al., 2020). The anaerobic conditions found in continuous flooding techniques are estimated to significantly impede NO_3_^−^ flux from nitrification while also promoting complete denitrification of any NO_3_^−^ to N_2_, resulting in minimal N_2_O emissions. Conversely, the AWD method involved a shift to more aerobic conditions and might have accelerated nitrification and NO_3_^−^ production increasing N_2_O production (Verhoeven et al., 2019; Islam et al., 2020). The integrative impact of soil moisture content in different rice production techniques and rice development stage could be ascribed to temporal CO_2_ flux fluctuation. The increased CO_2_ fluxes at panicle initiation stage were owing to increased carbon substrate and microbial activities (Swain et al., 2018). In comparison to continuously flooded systems, AWD technique mitigated global warming potential (GWP: CO_2_+CH_4_+N_2_O) by 45%–90% (Linquist et al., 2012).

### 4.3 Effect of different rice production techniques on soil health indicators and climate smartness index

The post-harvest soils of SRI-AWD technique had higher plant biomass that might result in secretion of root exudates and metabolites that improved water-soluble carbon (Thakur et al., 2011; Mandal et al., 2020; Wen et al., 2022). Generally, soil moisture regime of 60%–80% field capacity is best suitable for microbial activity (Linn and Doran, 1984; Zhang et al., 2020). Hence, MBC content was higher in SRI-AWD, DSR-AWD, and TPR-AWD as compared to FPR-CF. Tian et al. (2013) reported abundance of bacteria and AM fungi, and actinomycetes population decreased under continuous submerged situation. Anoxic flooding condition favored methanogenic archaea activities over alternate wetting and drying condition, whereas a reverse trend occurred in denitrifier’s activity (Lagomarsino et al., 2016). In SRI technique, organic manure was added to soil during land preparation, which could have improved microbial and enzyme activities (Lakshmi et al., 2019). The urease activity was higher under AWD condition as compared to CF, which could be referred to a decrease in oxygen diffusion rate (ODR) value (Naher et al., 2021). Similarly, β-glucosidase activities are primarily responsible for the conversion of low-molecular-weight carbon molecules to sugar in response to soil moisture regimes (Turner et al., 2002; Nayak et al., 2007; Watts et al., 2010). Continuous submerged soils had higher dehydrogenase activity than soils with alternately wet and dry. Low ODR was shown to be favorable for dehydrogenase activities in soil in previous studies (Wolińska and Bennicelli, 2010; Lakshmi et al., 2019).

The CSI presented here suggests universal improvements in the selection of climate-smart rice production techniques when compared with farmer practices. The approach to developing a CSI presented here offers a means for quantitatively measuring and comparing the combined mitigation, adaptation, and productivity properties of agricultural practices. The specific CSI presented is a suitable metric for contexts of climate-driven constraint relating to water stress like drought, changing rainfall patterns, increasing temperatures, and the socioeconomic condition of farmers. The selection of the indicators for the development of CSI was done by combining mitigation, adaptation, and productivity properties such as GHG, PFPN, IWP, EP, BC ratio, and yield of rice farming. SRI-AWD, DSR-AWD, and TPR-AWD had higher CSIs, which might be due to their higher yield potential, water productivity, nutrient use efficiency, global warming mitigation potential, and economics compared to continuous flooding rice production techniques (Yang et al., 2012; Fangueiro et al., 2017; Arenas-Calle et al., 2019). Linquist et al. (2015) and Tarlera et al. (2016) reported that in AWD trials, water savings and emissions reductions outweighed yield costs when compared with continuous flooding practices. Consequently, we cannot explain the climate smartness associated with rice establishment method, irrigation, and nutrient management without considering suitability, and the situations in which individual CSA pillars improve considerably with respect to others, or even at the expense of others, should be carefully considered, as CSA priorities may not be the same in all cases (Campbell et al., 2014; Lipper et al., 2014; Totin et al., 2018). This is important because rice is also threatened by sub-emergence, soil salinity, and high temperatures (Mohanty et al., 2013), which means that the meaning of “climate smart” may change. The CSI could offer an easy interpretation and a transparent measure of climate smartness.

## 5 Conclusion

The present research calculates a climate smartness index and then applies it to screening different rice production techniques based on their score. The CSI was constructed by taking relevant indicators such as grain yield, irrigation water productivity, nutrient use efficiency, energy productivity, global warming potential, and the economy, then normalizing and weighing those indicators, and finally calculating the CSI by aggregating indicator scores and weightage using additive methods. Among the climate-resilient rice methods, SRI-AWD is the best method in terms of CSI score. The approach provides a pragmatic solution to meet the several needs and advantages of adaptation progress monitoring, effectiveness, and reduced supply chain risks resulting from climate change. Even so, as this is still a preliminary research, more investigation and assessment in multiple years, multiple locations, and multiple climatic situations are needed to reduce the uncertainty in CSI assessments, particularly in terms of spatial and temporal variability in yield, GHG mitigation potential, soil health, water productivity, and profitability in large-scale agricultural systems.

## Data availability statement

The original contributions presented in the study are included in the article/supplementary material. Further inquiries can be directed to the corresponding author.

## Author contributions

AN laid and managed experiments. KM and CS collected data, analyzed, and wrote the first draft of manuscript. AN reviewed and edited the manuscript. The rest of the authors read the manuscripts and corrected where ever necessary. All authors have read and agreed to the published version of the manuscript.
